# Serious Notifications on Food Contact Materials in the EU RASFF

**DOI:** 10.3390/vetsci8040056

**Published:** 2021-03-29

**Authors:** Elias P. Papapanagiotou

**Affiliations:** Laboratory of Animal Food Products Hygiene-Veterinary Public Health, Department of Hygiene and Technology of Food of Animal Origin, School of Veterinary Medicine, Faculty of Health Sciences, Aristotle University of Thessaloniki, 54124 Thessaloniki, Greece; ipapapan@vet.auth.gr

**Keywords:** RASFF, serious, alert, border rejection, food contact materials, HFAA/ex-FVO, BTSF, EFSA, Codex Alimentarius Commission

## Abstract

Serious alert and border rejection notifications on food contact materials (FCM) retrieved from the RASFF database were analyzed for the first time regarding the period 2012–2019. The findings indicate that China was the main transgressor country for both types of notifications. Official controls were responsible for most FCM serious alerts (91.78%), and border rejection (90.82%) notifications. Another novelty proposed herein, is the criterion for “lag phases” (time from sampling to notification dates). Overall percentage distributions of lag phases, for all RASFF Member States, for the intervals of 0–50 days and 51–≥101 days, were 25.09% and 67.87% for serious alert notifications and 65.21% and 29.34% for serious border rejection notifications. Differences in percent shares of lag phase intervals were observed between the top-four notifying countries, indicating a lack of harmonization in timely reporting of serious alert and border rejection notifications for FCM. Migration of primary aromatic amines and of metals were the most frequently notified hazards overall in the period of analysis. A decreasing trend is observed in the two more recent biannual averages of serious alert notifications for primary aromatic amines and metals, while decreasing for metals but increasing for primary aromatic amines in serious border rejection notifications.

## 1. Introduction

Food contact materials (FCM) are all materials and articles intended to come into contact with food, such as packaging and containers, kitchen equipment, cutlery and dishes, also surfaces of instruments and machinery. These can be made from a variety of materials including plastics, rubber, paper and metal. The safety of food contact materials must be evaluated as chemicals can migrate from the materials into food. The materials must be manufactured in compliance with EU regulations, including Good Manufacturing Practices (GMP), so that any potential transfer to foods does not raise safety concerns, change the composition of the food in an unacceptable way or have adverse effects on the taste and/or odor of foods [1].

The EU Rapid Alert System for Food and Feed (RASFF) has its legal basis in Regulation 178/2002 [2]. Among the different types of RASFF notifications, the two most significant are alert and border rejection notifications. Alert notifications are submitted to the RASFF when a food, feed or food contact material presenting a serious risk to human health is on the market and when rapid action is or might be required by another member of the RASFF network. Alerts are notified by the member of the RASFF network that first detects the hazard and has taken steps to mitigate the risk (withdrawal from the market, recall from consumers, destruction, etc.). The alert notification ideally offers the other members of the RASFF network the necessary information to confirm whether the concerned product is on their market, so that they too can take the appropriate action. Border rejection notifications concern a transgression consignment of food, feed or food contact material that was refused entry into the EU for reason of a risk to human health and also for reason of a serious risk to animal health or to the environment if it concerns feed [3].

The role of the RASFF network is to act as an early warning tool between RASSF members to ensure the flow of valuable information between member states in order to promptly react when risks to public health are detected. The RASFF enables information exchange concerning measures aimed at the withdrawal of food or feed, including food contact materials from the market to be shared efficiently between the national food safety authorities of the EU-28, Iceland, Liechtenstein, Norway and Switzerland, the Commission, EFSA and the European Economic Area.

The international character of the RASFF is long-standing through its close collaboration with the International Food Safety Authorities Network (INFOSAN) of the World Health Organization (WHO), when a public health emergency of international concern (PHEIC) is identified. Since 2004, the INFOSAN has managed food safety events with international implications at the WHO. The INFOSAN Secretariat should be systematically informed via online database RASFF Window on the notifications and relevant follow-ups to: (a) a foodborne outbreak that has been linked to a food product that has been either imported from or exported to non-member country; (b) a multi-country foodborne outbreak that has been identified, but the source of the food product has not yet been determined; and (c) contaminated food, feed or food contact materials that have been identified as posing a serious health risk which has either been imported from a non-member country or has been exported to a non-member country [4].

Food contact materials play a significant and perhaps underestimated role in the food supply chain, in the general context of food safety. Consumers use kitchen utensils, tableware and cutlery on a daily basis, and it is usually children or infants that come into contact with these items by and large to consume their food. Because food contact materials are not chemically inert and thus may release very small amounts of substances into food, the EU has established legislation for their production and has posed restrictions, e.g., migration limits. Harmonized rules on the chemical safety of food contact materials at EU level could undoubtedly help to protect consumers’ health and to remove trade barriers [5]. Unfortunately, the vast majority of food contact materials are not subject to a specific EU legislation. Moreover, food contact materials legislation across the European Member States is not harmonized with regards to limits.

The European Union Reference Laboratory for Food Contact Materials (EURL-FCM) provides scientific and technical assistance to the EU and the Member States. It organizes inter-laboratory comparison exercises and conducts training courses for the benefit of National Reference Laboratories and of experts from developing countries. It is supported by a network of national reference laboratories [6].

The EU legislation pertaining to food contact materials includes the following: (i) Regulation (EC) No 1935/2004 [7] on materials and articles intended to come into contact with food and repealing Directives 80/590/EEC and 89/109/EEC; (ii) Commission Regulation (EU) 2018/213 [8] on the use of bisphenol A in varnishes and coatings intended to come into contact with food and amending Regulation (EU) No 10/2011 [9] regarding the use of that substance in plastic food contact materials; (iii) Regulation (EC) No 450/2009 [10] on active and intelligent materials and articles intended to come into contact with food; (iv) Regulation (EC) No 282/2008 [11] on recycled plastic materials and articles intended to come into contact with foods and amending Regulation (EC) No 2023/2006; (v) Directive 84/500/EEC [12] on the approximation of the laws of the Member States relating to ceramic articles intended to come into contact with foodstuffs; (vi) Directive 2007/42/EC [13] relating to materials and articles made of regenerated cellulose film intended to come into contact with foodstuffs; (vii) Regulation (EC) No 2023/2006 [14] on good manufacturing practice for materials and articles intended to come into contact with food; (viii) Regulation (EC) No 1895/2005 [15] on the restriction of use of certain epoxy derivatives in materials and articles intended to come into contact with food;and (ix) Directive 93/11/EEC concerning the release of N-nitrosamine and N-nitrosatable substances from elastomer or rubber teats and soothers [16].

More specifically, article 3 of the Framework Regulation 1935/2004 specifies the three key requirements on food contact materials namely that the migrates must not: ‘‘endanger human health”, ‘‘bring about an unacceptable change in the composition of the food” (unacceptably contaminate), and ‘‘bring about a deterioration in the organoleptic characteristics thereof”.

In a recent paper [17] the authors have analyzed all types of RASFF notifications (irrespective of their risk decision) related to food contact materials from 2007 to 2019 by testing the significance of the relation between the notified products’ origin and other variables (i.e., hazard and type of food contact materials, notification basis, notification type, risk decision) and providing insights to the European trade relations and practices. Their results portray a picture of serious, not serious and the undecided (in terms of risk decision) notifications. It should be noted here that this mode of interpretation of RASFF notifications (not according to risk decision) is also found in the RASFF annual reports. This approach is certainly an important but not per se risk-based for the assessment of RASFF notifications, hence, the attempt to use only the serious notifications in which a serious risk decision has already been allocated in the RASFF database, from 2012 onwards.

In view of the aforementioned, the aim of this paper was to focus for the first time exclusively on the serious alert and border rejection notifications with a view to highlight the overall serious risks posed to human health from food contact materials from 2012 to 2019. In this way, a truly risk-based approach to challenges from various hazard types pertaining to food contact materials that have been identified by the RASFF can be implemented. Additionally, lag phases (by the subtraction of sampling dates from notification dates) will be calculated for the first time for all serious alert and border rejection notifications on food contact materials. Finally, risk containment and risk mitigation measures are proposed, vis a vis the EU institutions’ functions.

## 2. Materials and Methods

A search in the EU Rapid Alert System for Food and Feed database (https://webgate.ec.europa.eu/rasff-window/portal/?event=searchForm&cleanSearch=1) retrieved the data used for this paper (accessed on 25 July 2020). The selection from the RASFF Portal was on the one occasion “Food Contact Materials” AND “serious” AND “alert notifications” and on the other occasion “Food Contact Materials” AND “serious” AND “border rejection notifications”, both for the period from 1 January 2012 till 31 December 2019. The following fields were selected: notifying country, country of origin, hazard category, year of notifications, type of notification, subject, risk decision, distribution status, classification basis and dates of sampling and dates of notification from the details of every single notification.

The subtraction of the sampling dates from the notification dates of each serious alert and border rejection notification enabled the calculation of the lag phases (in days). The lag phases have been grouped in specific time periods 0–10 days, 11–20 days etc., so as to encompass groups of days for a more comprehensive presentation of the results and as percent (%) shares of all serious alert (sA) or serious border rejection (sBR) notifications. A biannual average of the incidence of migration hazards has been chosen as a means to more accurately describe the various trends in sA and sBR notifications over the period between 2012 and 2019. All data were extracted in Excel files in the various combinations chosen, and descriptive statistical methods were applied.

## 3. Results

### 3.1. Serious Alert (sA) and Border Rejection (sBR) Notifications as a PercentShare of All A and BR Notifications on Food Contact Materials

In Table 1, the percent (%) share of sA and sBR notifications on food contact materials, over all the A and all the BR submitted in the RASFF database, is shown. On average, over the period from 2012 to 2019, 80.53% and 18.31% of all A and all BR notifications on food contact materials were serious, respectively (indicating a 4.39 times difference in occurrence frequency in favor of sA over sBR notifications), bearing in mind that the sA notifications were almost twice as many as the sBR.

With regards to the sA, a decreasing trend was noticed from 2016 onwards, while for sBR a decrease was only seen from 2018 onwards. The peak in the percent (%) share of sAover all A on food contact materials was noticed in 2013–2015. The year 2018 represented the peak in the percent (%) share of sBR over all BR notifications on food contact materials in the RASFF.

### 3.2. Serious Alert (sA) Notifications on Food Contact Materials

The first ever sA notification on food contact materials was notified by Germany on 10 August 2012 (with the date of sampling being 29 May 2012) and the subject was the migration of formaldehyde and of melamine from kitchen spoons from Slovakia. The twenty (of a total of 32, excluding Commission Services)RASFF member states (MS) that have submitted sA notifications were the following (in descending order): Germany (*n* = 51), Poland (*n* = 36), Belgium (*n* = 18), France (*n* = 15), Czech Republic and Slovenia (*n* = 14), Cyprus and Ireland (*n* = 10), Luxembourg and Austria (*n* = 7), United Kingdom (*n* = 5), Finland (*n* = 4), Slovakia, Estonia, Greece (*n* = 3), Latvia and Denmark (*n* = 2), Italy, Netherlands and Switzerland (*n* = 1). Collectively, the contribution of the two greatest contributors namely, Germany and Poland accounted for 42.02% of all such notifications.

The greatest part (91.78%) of all sA notifications were submitted as an official control on the market which signifies that the Competent Authorities (CA) of the respective MSs were responsible for sampling and analysis for the greatest part of food contact materials’ transgression notifications. The remaining 17 notifications were made on the basis of: (i) company’s own check (*n* = 8); (ii) consumer complaint (*n* = 6); and (iii) border control-consignment released (*n* = 3).

The WHO INFOSAN was included in more than half (56.52%) of all sA notifications in their “countries/organizations concerned” in their notification details. This finding concerns a significant number of countries (both within and outside the EU) which could potentially have been affected by a health-threatening incident involving food contact materials. Furthermore, a 14.97% of all sA notifications involved Commission Services (CS) in their “countries/organizations concerned” in their notification details.

China was the country of origin that has by and large most frequently been notified against withsA notifications on food contact materials in the EU RASFF database from 2012–2019. The majority (67.14%) of sA notifications implicated China as the country of origin; the latter being either directly (“from China”) in 33.81% or indirectly (“from China, via” another country) in 66.18%, as was stated in the subject of these notifications. This indirect way of China being notified as a country of origin is a finding of significance, since it obviously points out the fact that the faulty consignment had already gained entry into the EU from the country of origin (namely China) via another EU member state (which had acted as the first point of entry in the EU market). In the 92 sA notifications that stated in their subject “from China, via” another country, these countries were Poland, Germany, the Netherlands, Hong Kong, Slovakia, the Czech Republic, Spain, the United Kingdom, Lithuania and Sweden with the Netherlands having been mentioned in 16 (17.39%) such notifications. On the other hand, Poland was the notifying country that had notified the highest number (20/92 or 21.73%) of such notifications. Interestingly, there were seven notifications submitted against faulty consignments originating from China via Hong Kong either directly (*n* = 2) or indirectly, via another country (*n* = 5) and these were the following: (i) from China, via Hong Kong and via Spain, (ii) from China, via Hong Kong and via Italy, (iii) from China, via Hong Kong, via Spain and via Germany, (iv) from China, via Hong Kong and via Slovakia, (v) from China, via Hong Kong and via Slovakia.

#### 3.2.1. Distribution to Other Member Countries in sA Notifications on Food Contact Materials

The finding that in 64.25% (133/207) of all sA notifications a designation of “distribution to other member states” was reported in their distribution status, deserves additional attention. Another finding of interest was the actual number of countries involved in the distribution of faulty consignments especially when it exceeded 16 countries per notification (excluding INFOSAN, CS and country of origin when it was not mentioned as a distribution country). Twenty such notifications were identified, and this means that the “risk dispersion” was quite widespread. There were also examples of sA notifications that one could highlight, in which the number of countries involved in the distribution of the faulty consignment were 30 or more. Some of the aforementioned examples of such notifications made on the basis of official control on the market and having widespread distribution (and hence potentially extensive “risk dispersion”) to numerous other countries are the following having been notified by: (i) Germany on 2 October 2017, the subject being migration of formaldehyde and melamine from coffee cups from the Netherlands (30 distribution countries), (ii) France on 25 September 2018, the subject being migration of melamine from bamboo fiber cutlery from a tableware set for children from China, via Denmark (32 distribution countries), (iii) Switzerland on 27 November 2018,the subject being a high content of lead in and migration of lead from sets of oil and vinegar dispensing cruets from Portugal, via France (32 distribution countries), and (iv) Luxembourg on 11 June 2019, the subject being migration of melamine from melamine plates from China, via Denmark (30 distribution countries).

The conceptual definition of “risk dispersion” in effect concerns both the actual number of countries that the transgression consignment was destined for distribution, but equally importantly the global dimensions of such a distribution, namely, countries from Europe, together with countries from the Caribbean Sea, from Latin America, from North America, from Africa, Asia, Oceania/Australia (for example Lebanon, New Zealand, South Africa, United States, Martinique, Uruguay, Peru, Chile, Morocco, South Korea, Thailand, Taiwan, Singapore, Australia, Israel, Turkey, United Arab Emirates, Georgia, Russia, Ukraine, Japan, Philippines were included, in the abovementioned four notifications).

#### 3.2.2. Lag Phases (from Sampling Dates to Notification Dates) in sA Notifications on Food Contact Materials, Signifying Efficacy of Timely Reporting by the Member Country to the RASFF

An innovative criterion proposed herein, namely the lag phases, was deduced from the sA notifications details in the RASFF database for the first time and was calculated by the subtraction of the sampling dates from the notification dates (in days). This difference should in principle be as small as possible, meaning that the issuing of a notification should be as close as can be practically feasible to the sampling date for obvious reasons aiming to the safeguarding of Public Health. Since the majority of the sA notifications were designated as an official control on the market, the lag phases thus represented the competent authority’s efficacy to manage each sample from beginning to end.

Some shortcomings in the reporting of the notifications were found and these pertained to: (i) notifications with no sampling date (*n* = 15); (ii) notifications with an inverse date of sampling, the sampling date being later than the notification date (*n* = 4); and (iii) sampling date given as 1 January 1970 (*n* = 1). Regarding the aforementioned notifications that had these shortcomings, it is obvious that lag phases could not be estimated. The percent (%) share of lag phases (from sampling to notification dates) in sA for all MS as a whole and for the top four MS that have notified the highest numbers of sA, is shown in Table 2.

The percent (%) share of sA notifications as an overall value for all EU RASFF MS was collectively calculated, with lag phases of: (i) 0–30 days was 8.19%, (ii) 31–50 days was 16.90%, (iii) 51–≥100 days was 65.21%. No lag phases could be calculated for the notifications that presented shortcomings and this represented 9.65% of all sA notifications on food contact materials.

The notifying country that has submitted by far the most (n = 23) sA notifications with a lag phase of ≥101 days was Germany, and these notifications included: (i) eight on migration of metals [e.g., nickel, (lead and cobalt), iron, (arsenic, cadmium and antimony), cobalt, nickel, lead, (cadmium and lead)]; (ii) seven on the migration of formaldehyde; (iii) six on the migration of primary aromatic amines; (iv) one on the migration of melamine; and (v) one on high content of DEHP-di(2-ethylhexyl) phthalate. Regarding the EU RASFF MS that has notified the majority of sA notifications, namely Germany, it should be highlighted that it has achieved a lag phase of 0–30 days in just 1.96% of cases with zero percent (%) share in the 0–10 days and 11–20 days of all its sA notifications, which is a finding that presents a challenge. Contrary to this finding, the second ranking EU RASFF MS, Poland, has succeeded in attaining for the same lag phase a significant share (24.99%) of its notifications. On the other hand, both countries show their highest percent (%) share in specific lag phases e.g., for Germany 45.09% in the ≥101 days lag phase and Poland 36.11% in the 51–100 days lag phase. This only exemplifies the variation in lag phases between these two top-ranking EU MS. Belgium and France had a percent share of the 0–30 days lag phase of only 5.56% and 13.34%, respectively, whereas in the 51–≥101 days lag phase their percent (%) share was 38.89% and 53.34%, respectively. The highest percent (%) share of sA of Belgium and France was found to be in the lag phase of 51–100 days for both MS.

Clearly, if one were to utilize the overall (all EU RASFF MS collectively assessed) values of percent (%) share of lag phases as a reference and compare the top four MS with that (and for all MS by the same token), one would discover a large variation in almost all lag phases between MS. Additionally, the range of the percent (%) share in every single lag phase of the top four MS was rather large. For example, in the top four MS, the range of the percent (%) share of lag phases of 21–30 days, 31–40 days and ≥101 days was 0–19.44% (compared to an overall value of 5.79%), 3.92–22.22% (compared to an overall value of 5.31%), and 0–45.09% (compared to an overall value of 18.84%), respectively.

#### 3.2.3. Hazards Identified in sA Notifications on Food Contact Materials

As shown in Table 3, the overall average percent (%) share of the hazards (over the period 2012–2019) identified in the sA notifications on food contact materials (in a descending order of incidence percent share) were the following:

1. migration of primary aromatic amines,
2. migration of metals, which are here presented in detail: cadmium (*n* = 5), cadmium and lead (*n* = 25), cadmium and cobalt (*n* = 4), arsenic, cadmium and antimony (*n* = 1), cadmium zinc and aluminum (*n* = 1), arsenic and aluminum (*n* = 1), aluminum (*n* = 5), lead (*n* = 12), lead and cobalt (*n* = 1), chromium and lead (*n* = 1), cobalt (*n* = 3), nickel (*n* = 2), iron (*n* = 1),
3. miscellaneous which are represented by (i) migration of benzene; (ii)(risk of) breakage of bottles etc.; (iii) migration/high content of DEHP-di(2-ethylhexyl) phthalate; (iv) suffocation risk; (v) risk of mouth injury; (vi) too high levels of overall migration; and (vii) migration of p-tert-butylbenzoic acid (PTBBA), high content of DPHP-di(2-propylheptyl) phthalate, migration of BBP-benzyl butyl phthalate, and migration of N-nitrosatable compounds,
4. migration of melamine,
5. migration of formaldehyde and of melamine, and
6. migration of formaldehyde.

The combined average percent (%) share of primary aromatic amines (PAA) and metals amounted to 61.29% of all hazards over the 8-year period under examination.

In the same way that one can deduce valuable information on the lag phases that all RASFF MS overall have achieved for sA notifications for all hazards related to food contact materials, one could also determine the lag phases for the specific hazards identified from the same dataset. In this way, the lag phases of the specific sA notifications on FCM that implicated migration of PAA and migration of metals as the hazards identified, were 79.13 ± 43.27 days and 73.69 ± 48.41 days, respectively.

Furthermore, the same dataset of lag phases on food contact materials could be used to evaluate the MS-specific responsiveness in notifying against each specific hazard implicated. For example, Germany showed an overall lag phase of 103.37 ± 64.68 days for metals and 115.29 ± 13.65 days for PAA, whereas Poland showed an overall lag phase of 40.94 ± 24.55 days for metals and 63.00 ± 37.16 days for PAA.

More specifically, as shown in Table 3, a clear decreasing trend in the average percent contribution of migration of PAA has been noticed in the last two biannual periods, namely 2018–19 and 2016–17, when compared to the earlier two periods. With regard to the migration of metals, only in the latest biannual period has a decreasing trend been noticed compared to the previous one. Another example of a significant decreasing trend was noticed in the group collectively named “miscellaneous” over the 2018–19 period compared to the previous one. On the other hand, a finding worth noticing is the significant increasing trend in the migration of formaldehyde and of melamine in the last biannual period compared to the previous one.

### 3.3. Serious Border Rejection (sBR) Notificationson Food Contact Materials

As shown in Table 1, the proportion of serious over all types of risk decision (combined serious and not serious and undecided) BR notifications on food contact materials from 2012 until 2019 was 18.31%, which is rather low, especially when compared to the respective percent found in sA notifications. The first ever sBR notification was notified by Italy on 20 July 2012 (with the date of sampling being 21 June 2012), with the subject being the migration of nickel and of manganese from a tray for air flow electric ovens from China. The fifteen RASFF MS (of a total of 32, excluding Commission Services) that have submitted the highest number of sBR notifications on food contact materials are the following (in descending order): Italy (*n* = 32), United Kingdom (*n* = 18), Finland (*n* = 16), Germany (*n* = 12), Spain (*n* = 9), Netherlands (*n* = 4), Poland (*n* = 4), France (*n* = 4), Greece (*n* = 2), Croatia (*n* = 2), Belgium (*n* = 2), Norway (*n* = 1), Cyprus (*n* = 1), Ireland (*n* = 1)and Slovenia (*n* = 1). Interestingly, Italy and the United Kingdom together have contributed 45.87% of all sBR notifications. All sBR notifications contributed to RASFF by the United Kingdom were on PAA originating from China, except for two that originated from China via Hong Kong.

The majority of all sBR notifications (90.82%) were submitted as a border control-consignment detained, which means that the competent authorities were responsible for sampling and analysis of the majority of food contact material transgressions that were refused entry into the EU market. The remaining notifications were made on the basis of: (i) border control-consignment under customs (*n* = 9) and (ii) company’s own check (*n* = 1).

In contrast to the respective finding in sA notifications, the WHO/INFOSAN was not included in any of the sBR notifications in their “countries/organizations concerned” in their notification details, whereas the Commission Services (CS) were only included in their “countries/organizations concerned” in their notification details, on just eight occasions (7.33%).

China as the country of origin (like in sA notifications) dominated the picture in sBR notifications on food contact materials. Almost all sBR notifications implicated China as the country of origin (94.49%), either directly (“from China”) in 89.32% or indirectly (“from China, via” another country) in 10.67%. With regards to the latter, ten were from China, via Hong Kong and one from China, via Switzerland. The notifications that mentioned the origin of the transgression consignment as being “from China” were notified to the EU RASFF by the following countries: Italy (*n* = 26), United Kingdom (*n* = 16), Finland (*n* = 14), Germany (*n* = 12), France (*n* = 3), Spain (*n* = 7), Poland (*n* = 4), Netherlands (*n* = 3), Greece (*n* = 2), Cyprus, Belgium, Ireland, Croatia and Slovenia (*n* = 1).Apart from China, only six notifications were found implicating other countries of origin, namely, three from Hong Kong, and one each from Thailand, Russia and Ukraine.

#### 3.3.1. Distribution to Other Member Countries in sBR Notifications on Food Contact Materials

The exact opposite of the situation described for sA notifications concerning “risk dispersion” is portrayed for sBR notifications, since not even one case was found to exist with ≥16 countries involved in the distribution list of faulty consignments. The vast majority of sBR notifications had only 2–4 distribution countries (excluding Commission Services) in their “countries/organizations concerned” in the notification details.

#### 3.3.2. Lag Phases (from Sampling Dates to Notification Dates) in sBR Notifications on FCM, Signifying Efficacy of Timely Reporting by the Member Country to the RASFF

Asin sA notifications, the same procedure was followed for sBR notifications in order to display the lag phases between the sampling and the notification dates. A few shortcomings in the reporting of the BR notifications were also recorded, and these pertained to notifications with no sampling date (*n* = 3). The percent (%) share of lag phases for all MS as a whole and for the four MS that have notified the highest numbers of sBR, is shown in Table 4.

The collective percent (%) of sBR notifications that have a lag phase from sampling to notification dates of (i) 0–30 days was 45.87%; (ii) 31–50 days was 22.01%; (iii) 51–≥100 days was 29.34%. Obviously, lag phases could not be calculated for the notifications that presented shortcomings and this represented 2.75% of all sBR notifications. It is obvious that the most challenging lag phase being the latter does not seem to represent a serious shortcoming with regards to the BR notifications.

Regarding the EU RASFF MS that notified the majority (29.35%) of all sBR notifications, Italy, it should be highlighted that it has succeeded in achieving a lag phase of 0–30 days for the 84.37% of all its notifications, which is a most remarkable and at the same time promising finding. An excellent example to illustrate the aforementioned comment is a notification (with a notification date of 29 May 2013 and a sampling date of 28 May 2013) that was notified by Italy, the subject being the migration of lead from stainless steel oil pots from China, in just one day. Contrary to that finding, the second EU RASFF MS in ranking (in numbers of such notifications), namely the United Kingdom, has succeeded in attaining for the same lag phase of 0–30 days in only 44.43% of all its notifications. It is evident that not all EU RASFF MS seem to have the same percent share of lag phases in their sBR notifications just as was found in sA notifications.

Another striking difference between Italy and the United Kingdom can be seen in the 0–10 days lag phase, in which the former shows a 3.3 times higher percent share in that lag phase (18.75% vs. 5.55%).

Finland and Germany had a percent share of the 0–30 days lag phase of 18.75% and 58.33%, respectively whereas in the 51–≥101 days lag phase their respective percent (%) share was 25.00% and 41.66%, respectively. In accordance with the sA, if the overall (all EU RASFF MS collectively assessed) values of percent (%) share of lag phases for sBR were used as a reference for comparison with the respective top four MS values, a large variation in almost all lag phases between MS would be revealed, again with the range of the percent (%) share in every single lag phase between the top four MS being rather large. For example, the range of the percent (%) share of lag phases of 11–20 days, 41–50 days and ≥101 days was 0–34.37% (compared to an overall value of 18.34%), 0–31.25% (compared to an overall value of 13.76%), 0–22.22% (compared to an overall value of 11.00%), respectively.

#### 3.3.3. Hazards Identified in sBR Notifications on Food Contact Materials

In Table 5, the overall average percent (%) share of the hazards (over the period 2012–2019) identified in the sBR notifications on food contact materials (in descending order of incidence percent share) are shown and these were the following:

1. migration of primary aromatic amines,
2. migration of metals which are here presented in detail: Cadmium (*n* = 5), Cadmium and lead (*n* = 5), Lead (*n* = 6), Nickel (*n* = 3), Nickel and manganese (*n* = 2), Lead and Nickel (*n* = 1), Chromium (*n* = 2), Chromium, nickel and manganese (*n* = 2), Chromium lead and manganese (*n* = 1), Cadmium and copper (*n* = 1), Manganese (*n* = 1).
3. migration of formaldehyde,
4. migration of formaldehyde and of melamine,
5. miscellaneous which are represented by (i) plastic fragments (fibers) in ateflon basket for barbeques; (ii) too high levels of overall migration from cutlery; (iii) DEHP-di(2-ethylhexyl) phthalate, and
6. migration of melamine.

The lag phases dataset for sBR notifications on food contact materials was also used to assess the overall time responsiveness of notifications in a hazard-specific manner. The lag phases of the specific notifications that implicated PAA and metals as the hazards identified, were 68.31 ± 94.97 days and 33.79 ± 25.69 days, respectively.

Furthermore, the same dataset of lag phases for sBR notifications could be used to evaluate the MS-specific responsiveness to notifying against each specific hazard implicated. For example, Italy showed an overall lag phase of 21.13 ± 11.68 days for metals and 23.22 ± 11.94 days for PAA, whereas the United Kingdom showed an overall lag phase of 76.28 ± 84.98 days for PAA only because all of its notifications were on PAA.

The combined average percent (%) share of PAA and metals amounts to 84.08% of all hazards identified in the sBR notifications over the 8-year period examined. More specifically, as shown in Table 3, an increasing trend in the average percent (%) contribution share of migration of PAA has been noticed in the last biannual period, namely 2018–19 compared to the previous three periods. With regards to the migration of metals in the latest biannual period a sharp and remarkable decreasing trend has been noticed compared to the previous one. On the other hand, a finding worth highlighting is the increasing trend in the migration of formaldehyde in the last biannual period (2018–19), compared to the previous one.

## 4. Discussion

An attempt was made in this paper to exclusively analyze the sA and sBR notifications on FCM in the EU RASFF, for the first time in bibliography. This attempt was justified by the rationale that the serious notifications are the ones that entailed true and unequivocal substantiated risks to human health over the years from 2012 until 2019, and as such deserved to be given the highest priority as far as their thorough interpretation is concerned, for the benefit of EU institutions and consumers at large. The renowned EU Institutions (e.g., RASFF, HFAA/ex-FVO, BTSF, JRC) have enormously contributed for many years towards providing the necessary scientific knowledge and background information to European policy makers in the development and implementation of EU legislation on FCM. A number of new findings of this paper could potentially provide the stimuli to instigate additional action on the part of the EU Institutions in order to further improve the current situation concerning serious FCM transgressions.

It has become evident from the analysis of the data herein that the RASFF MS’ competent authorities were responsible for the controls from sampling until reporting of the transgressions, for more than 9 out of 10 sA and sBR notifications. Hence, their timely responsiveness via the calculation of lag phases (from sampling dates to notification dates) in both sA and sBR notifications in food contact materials was deemed necessary and was used for the first time in bibliography.

The examination of the lag phases has revealed that great variation exists in the percent (%) share of all the lag phases calculated for both sA and sBR notifications on food contact materials when each MS was compared against the respective set of values of the overall lag phases (all EU RASFF MS collectively assessed), but also when compared to one another (as exemplified in the top four MS examined here).

The 0–50 days lag phase of all EU RASFF MS examined collectively was 25.09% and 67.87% for sA and sBR notifications, respectively. On the other hand, the 51–≥101 days lag phase of all EU RASFF MS examined collectively was 65.21% and 29.34% for sA and sBR notifications, respectively. This finding points out that the reaction time in sBR notifications was by far shorter than that of sA notifications.

Lag phase heterogeneity was evidenced between the four highest notifying countries for both types of notifications, for example, in sA notifications a rather large range of percent share of lag phases was evidenced, such as in the 21–30 days (0–19.44%), the ≥101 days (0–45.09%), etc. Similarly, in sBR notifications in the top four notifying MS, an equally large range of percent share of lag phases was evidenced, such as in the 0–10 days (0–18.75%), the 11–20 days (0–34.37%), the 41–50 days (0–31.25%), etc. It is not surprising that EU RASFF MS differ with regards to their percent share of lag phases in their sA and sBR notifications, and this highlights a lack of harmonization. However, other relevant parameters should also be taken into consideration e.g., structure of the organization of official controls, number of laboratories, number of competent personnel, complexity and length of analytical methods etc.

This heterogeneity could be ameliorated by fact-finding missions by the European Commission’s HFAA/ex-FVO in view of attaining some degree of harmonization between MS by and large, with regards to FCM notifications’ timely reporting. It has become apparent that for both types of notifications examined herein, examples of EU RASFF MS that have attained excellent percent share of lag phases (e.g., Italy in sBR) do actually exist and their experience perhaps would be most valuable to other MS. Additionally, a comparison of lag phases attained by each EU RASFF MS against the overall average of all EU RASFF MS collectively, could perhaps represent a starting point for a novel approach or modus operandi, vis a vis harmonization in the timely submission efficiency of sA and sBR notifications on food contact materials.

Furthermore, lag phases for specific individual hazards identified in the sA and sBR notifications have been estimated. Thus, it has been shown that the average lag phases for the migration of PAA in both types of notifications do not differ that much (10.82 days), being shorter in the sBR notifications when compared to sA. On the other hand, the average lag phases for migration of metals differ much more (39.90 days), being shorter in the sBR notifications, pointing to a much faster response time for metals in comparison to PAA. Again, differences in analytical methods used and other parameters should be taken into consideration when addressing such issues.

Additionally, the lag phases have been used to estimate each MS’s individual capacity to timely respond to each specific hazard identified in food contact materials by submitting serious notifications to the RASFF. For example, in sA notifications Poland attained much shorter lag phases for both metals and PAA migration transgressions than Germany. By the same token, regarding sBR notifications, Italy attained much shorter lag phases in PAA migration transgressions than the United Kingdom.

With regards to the most frequently implicated hazards, overall these were the migration of PAA and migration of metals, representing 61.29% and 84.08% of all sA and sBR notifications, respectively. So, the migration of PAA and metals should be the specific hazards in food contact materials that the regulatory efforts should focus on. The “risk dispersion” of transgression consignments was generally far greater in A than in BR notifications, due to the much higher numbers of distribution countries in the former.

Another interesting finding from analyzing the dataset of lag phases was the ability to assess each individual MS’s values in sA compared to sBR notifications on food contact materials. For example, Germany seems to have attained for the lag phases of 0–40 days, and 0–100 days for sA vs. sBR notifications, 5.88% vs. 58.33% and 47.05% vs. 91.66%, respectively. Also, shortcomings in the sBR notifications details were not found at all, whereas in sA these represented 7.84% of all sA. Consequently, it seems that even the same MS has huge variation in the percent share of lag phases of the sA vs. the sBR it has notified.

The migration of metals is a finding worth highlighting, since cases of multiple metals identified in one single notification have been found in the RASFF dataset analyzed. As was shown in this paper, the trend for the migration of certain hazards (formaldehyde, melamine, metals, miscellaneous) in sA and sBR notifications as a metric of biannual average follows the same pattern; the former two showing an increasing trend whereas the latter two a decreasing one. A diversified trend exists for the migration of PAA and for the migration of formaldehyde and of melamine in sA (marginally decreasing and increasing trend, respectively) and in BR (increasing and steady trend, respectively) notifications when viewed as a biannual average. The migration of more than one metals effectively represents a good example of mixture toxicity that was revealed herein, for which risk assessment (RA) should be implemented accordingly. According to the results concerning the migration of metals identified in sA and sBR notifications, the former encompasses a larger compilation of different metals (lead, cadmium, aluminium, nickel, cobalt, arsenic, iron, chromium, antimony, zinc), in comparison to the latter (lead, cadmium, manganese, nickel, copper and chromium).

The serious risk for humans involved in food contact materials and originating from metals should be added on top of the serious risk for humans involved in food, when metals may also be involved in the latter. Other sources may contribute to actual human exposure, not only the substance’s migration from food packaging and consequently, the true exposure may be underestimated and safe exposure levels may be exceeded. As a result, RA for metals in food and in food contact materials combined would give a more accurate estimate of the actual overall risk to humans. Exposure assessment is a key issue in the food contact materials RA since many of the products incriminated for serious notifications could target children/infants, which is a highly vulnerable population group and mostly come into contact with utensils and kitchenware related to food contact material hazards.

In a recent excellent paper [18], it was stated that improving RA in the area of food contact materials will lead to a greater protection of public health by reducing exposure to hazardous chemicals. For the RA of a chemical, information on toxicological properties, as well as human exposure, is needed. In addition, humans are not only exposed to food contact chemicals (FCCs), but also to chemicals from other sources [19]. In the latter paper, mixture risk assessment (MRA) was discussed with exposure assessment being a major part of it, that required better modeling to predict combined exposures and correlated exposures but also for wider data collection and surveillance. This presents a clear challenge for toxicology (scientific tier) in the future and for new legislation (regulatory tier) covering such complex matters.

By far, the third country reported as being the country of origin for the vast majority of transgressions in both sA and sBR notifications was China, and the most often cited hazards in both types of notifications originating from China (either directly or indirectly via another country) were the migration of PAA and migration of metals. The indirect way of transgression consignments reaching the EU market should be thoroughly re-visited. With regards to China, Commission Regulation (EU) No 284/2011 [20] was put in force following the missions of the Food and Veterinary Office (FVO) to China and Hong Kong in 2009, where serious deficiencies were identified in the official control system regarding plastic food contact materials intended for importation into the Union and large quantities of controlled polyamide and melamine plastic kitchenware originating in or consigned from China and Hong Kong still not fulfilling the requirements of Union legislation at the time of publication of this Regulation. In short, Regulation 284/2011 which came into effect on the1 July 2011, applies to polyamide and melamine plastic kitchenware originating in or consigned from China and Hong Kong. From that date, imports have to be accompanied by a declaration and a laboratory report demonstrating compliance with the limits set out in the legislative text on the release of PAA and formaldehyde. The controls include documentary checks on all consignments and identity and physical checks including laboratory checks of 10% of consignments.

The WHO/INFOSAN was included in more than half of the sA notifications’ “countries/organizations concerned” list, clearly indicating that these were rather important incidents that required international risk communication and collaboration. The same does not hold true for sBR notifications, where none such case was found to exist. So, it is the sA notifications of the EU RASFF that seem to carry a higher possibility of potential international risk concerning FCM, and ideally an effort should be made towards enhanced/intensified controls by MS but also towards a faster response time for sA notifications on the part of each MS. These actions coupled with the continuation of timely notification of sBRs (as shown herein), reflecting on the excellent performance that the EU MS have shown in their border inspection/control posts by effectively refusing entry to faulty consignments concerning food contact materials, and could pave the way for a more resilient EU stance.

Nevertheless, the findings revealed from the analysis of the RASFF database should be carefully interpreted, since the RASFF system provides information only on non-compliance data, so the percent (%) of compliance is not known and also no volumes of consignments are given in the details of each notification. These limitations should always be taken into consideration, and a most careful and thorough analysis of the RASFF data should be performed.

The EU through its esteemed institutions has been contributing towards the protection of the consumer against food contact materials associated risks for many years. A number of additional actions could possibly add on the EU’s options for action and these are shown below in the form of proposals for measures directly relevant to specific Institutions of the EU in order to minimize the numbers of sA and sBR notifications appearing in the EU RASFF in the future.

The former FVO (now called Health and Food Audits and Analysis, HFAA) has realized only two audits in China for food contact materials, one in 2007 (report 2007-7572) and another one in 2009 (report 2009-8156). None has been carried out ever since, regardless of the fact that sA and sBR notifications implicating China have been steadily notified in the EU RASFF from 2012 to 2019, as shown herein. The number of notifications was a criterion at the time (when no serious risk decision was available for notifications in the RASFF database), but here a proposal is put forward to take into account the numbers of serious notifications for FVO visits. In view of the high number of RASFF notifications, the European Commission decided to undertake a mission to China in 2009 to follow up the recommendations made in report SANCO 7572/2007 [21] that identified issues regarding food contact materials from China, mainly relating to the migration of primary aromatic amines from nylon kitchen utensils, the migration of chrome and cadmium from stainless-steel kitchenware, the migration of lead and cadmium from ceramic articles, the migration of formaldehyde from melamine kitchenware, total migration, and the migration of phthalates.

The second mission of the FVO in China for FCM which took place in 2009 [22], in its audit report stated that: “for food contact materials exported to the EU via Hong Kong, the procedure is as follows: some food contact materials exporters declare that these products are going to be exported only to Hong Kong. Therefore, these products are only tested on the basis of Chinese national standards, which in some cases mean that the products (e.g., nylon kitchenware) are not tested for compliance with certain EU requirements, such as PAAs. According to the RASFF database, a significant number of food contact materials consignments manufactured in China were exported to the EU via Hong Kong”. One FVO mission was realized in Hong Kong on food contact materials and that led to the publication of the audit report 2009-8158 [23], which revealed that the Hong Kong authorities had no legal basis for food contact materials control transited from mainland China via Hong Kong into EU even though these products were not controlled to EU standards in China. Given the then contemporary status it was not possible for Hong Kong authorities to implement export controls.

All FVO audits concerning food contact materials realized from 2007 until 2018 have exclusively targeted EU MS (three in Portugal, two in Lithuania and Latvia, and one each in Slovakia, Hungary, Sweden, Belgium, Austria, Italy, Bulgaria, Poland, the Czech Republic, Malta, Romania, Slovenia, the United Kingdom, Greece, Finland, Germany, Denmark and France) except for the three aforementioned missions realized in third countries namely, two in China and one in Hong Kong. A concise planning for more audits to China and Hong Kong on FCM could help to significantly lower RASFF notifications and limit the possibility of risk dispersion of hazards pertaining to FCM not only inEurope, but to many other countries around the world as well. To that end, a logical proposal would be for the EU to act in a proactive manner and to establish a permanent HFAA/ex-FVO office in China to examine full compliance with EU standards before export is allowed. The US FDA (Food and Drug Administration) has long sinceestablished offices in various parts of the world, namely Europe, China, India and Latin America [24].

The EU Better Training for Safer Foods (BTSF) program is an initiative of the DG Health and Food Safety, managed by the Consumers, Health, Agriculture and Food Executive Agency (Chafea). The main purpose of the BTSF non-EU program is to help developing countries set food safety systems and animal health conditions to a level that contributes to their economic development, while also providing the conditions for safe food production. The courses offered by the BTSF non-EUdo not include food contact materials for the time being [25]. Provided the food contact materials were included in such an internationally acknowledged excellent series of courses, both manufacturers and government officials of the third countries, would undoubtedly benefit enormously, leading to further economic development and the provision of safer products.

The European Food Safety Authority (EFSA) is not mandated with the authorization of food contact materials. The EFSA evaluates the safety of substances used in food contact materials including active and intelligent materials. EFSA also evaluates the safety of recycling processes for recycled plastics used in food contact materials [26]. EFSA’s scientific advice is available in the adopted scientific opinions of the Panel on Food Contact Materials, Enzymes and Processing Aids (CEPPanel). Grob in 2019 [27] has very rightly advocated that for food contact materials the contribution of the EFSA to the RA of migrating substances is small, and concluded that best use should be made of the excellent scientific tools elaborated by EFSA, primarily through an improved mandate from the European Commission as part of a more effective regulation for FCM.

The EU as a supranational organization is a member of other international scientific bodies such as the Codex Alimentarius Commission (CAC) of the FAO/WHO which has not yet been involved in setting standards for food contact materials. Perhaps, in the future, a possible involvement of the CAC in food contact materials could show the way for a global concerted action towards this safety issue. More specifically, work could potentially focus on implementing RA for the various hazards entailed alone and in combination (e.g., metals). A possible inclusion of food contact materials in the Codex Committee on Contaminants in Foods (CCCF) could perhaps be envisaged since food contact materials-related hazards are within the remit of this Committee [28]. This step would hopefully initiate a roadmap for the inclusion of food contact materials-related hazards/substances in the context of food safety by: (a) establishing permitted maximum levels; (b) preparing priority lists for risk assessment by the Joint FAO/WHO Expert Committee on Food Additives (JECFA); and (c) considering methods of analysis and sampling, etc. The CAC in its documentCXS 193-1995 [29] has discussed the following chemicals with reference to food contact materials: (i) acrylonitrile (monomer) with a guideline level (GL) of 0.02 mg/kg (provisional acceptance in 1984); (ii) Vinylchloride monomer with a guideline level (GL) of 0.01 mg/kg (provisional acceptance in 1984) and (iii) melamine with a toxicological guidance value (TDI) of 0.2 mg/kg b.w. (2008).

In the JRC (Joint Research Center) Science for Policy Report [30] it was concluded that “overall, the entire sector of food contact materials suffers to a certain extent from the current situation, which exhibits a lack of harmonization of materials listed under the framework regulation and is the object of issues relating to mutual recognition”. In the same report the following were stated: “Not all MSs regulate the same materials. Different MSs regulate different materials. The lack of official or agreed methods exacerbates the difficulties for industries to show compliance. It also makes enforceability more difficult for official controls. The absence of criteria in turn affects the controls for given materials and the reporting in the RASFF on the part of the control authorities”. The EU rules on food contact materials can be of general scope, i.e., apply to all food contact materials or apply to specific materials only. EU law may be complemented with member state national legislation if specific EU rules do not exist [31].

## 5. Conclusions

The combined implementation of the two proposed novel criteria with which to evaluate the RASFF database, namely lag phases (from sampling dates to notification dates) and targeted evaluation of sA and sBR notifications, have produced a rather broad spectrum of new information. This information originating from RASFF sA and sBR notifications on food contact materials can be summarized in the following three tiers: (i) the overall lag phases of all EU RASFF MS combined, concerning all hazards collectively; (ii) the lag phases of the top four notifying EU RASFF MS individually, concerning all hazards collectively, and (iii) the lag phases of each of the top four notifying EU RASFF MS individually, concerning specific hazards. Furthermore, another proposed option would be to assess the lag phases of each MS individually concerning specific hazards related to food contact materials on a yearly basis to evaluate long-term responsiveness consistency.

A series of EU-wide fact-finding missions could help to identify the specific conditions existing in each EU RASFF MS and by following the best practices used in some MS, facilitate the implementation of harmonization in lag phases foreach hazard identified in sA and sBR notifications regarding food contact materials, and also effectively increase the percent (%) share of shorter lag phases over longer ones for each MS. Additionally, future missions should be realized in China, the country most often notified against in sA and sBR notifications.

A holistic RA could be envisaged, to encompass the same hazard found in food and food contact materials, with metals being an excellent example to start with (especially when multiple metals are found to be present simultaneously, as was the case in food contact material sA and sBR notifications).

In conclusion, the protection of public health from food contact materials related hazards deserves the length and breadth of scientific and legislative consideration that food has received thus far, for the benefit of consumers’ health.

## Figures and Tables

**Table 1:** Yearly distribution and percent (%) share of serious alert (sA) and serious border rejection (sBR)notifications overall A and BR notifications on food contact materials in the EU RASFF from 2012 until 2019.

Year	All A Notifications	sA	sA/all A (%)	All BR Notifications	sBR	sBR/All BR (%)
**2012**	38	10	26.32	126	8	6.35
**2013**	23	22	95.65	152	17	11.18
**2014**	23	22	95.65	104	13	12.50
**2015**	24	23	95.83	82	9	10.98
**2016**	27	23	85.19	61	12	19.67
**2017**	34	30	88.24	45	8	17.78
**2018**	48	40	83.33	52	21	40.38
**2019**	50	37	74.00	76	21	27.63
**sum**	267	207		698	109	
**Average ± s.d.**	33.38 ± 11.06	25.88 ± 9.55	80.53 ± 23.18	87.25 ± 37.50	13.63 ± 5.45	18.31 ± 11.07

s.d. = standard deviation.

**Table 2:** The percent (%) share of lag phases (from sampling to notification dates) in serious alert (sA) notifications on food contact materials from the RASFF database (from 2012 until 2019) for all MS as an overall valueand the top four member states (MS) that have contributed the highest numbers of sAnotifications.

	Percent (%) Share of sA Notifications				
Lag phases (Days)	All RASFF MS (Overall)	Germany (DE)	Poland (PL)	Belgium (BE)	France (FR)
**0–10**	0.96	0	0	5.56	6.67
**11–20**	1.44	0	5.55	0	0
**21–30**	5.79	1.96	19.44	0	6.67
**31–40**	5.31	3.92	8.33	22.22	6.67
**41–50**	11.59	5.88	19.44	22.22	20.00
**51–100**	46.37	35.29	36.11	38.89	46.67
**≥101**	18.84	45.09	5.55	0	6.67
**No sampling date recorded**	7.24	5.88	2.77	11.11	6.67
**Inverse date**	1.93	1.96	2.77	0	0
**Sampling date given as 1 January 1970**	0.48	0	0	0	0

**Table 3:** Percent (%) share of the hazards identified in sA notifications in food contact materials (FCM) on a yearly basis (from the RASFF database between 2012 and 2019) and their biannual and overall averages.

Percent (%) Share of the Hazards Identified in sA on FCM in RASFF (2012–2019)						
Year	Migration of Primary Aromatic Amines	Migration of Formaldehyde	Migration of Formaldehyde and of Melamine	Migrationof Metals	Migration of Melamine	Miscellaneous
**sA notifications**						
**2012**	30	10	20	30	0	10
**2013**	50	9.09	0	31.81	4.54	4.54
**2012–13 average ± s.d.**	40.00 ± 14.14	9.55 ± 0.64	10.00 ± 14.14	30.91 ± 1.28	2.27 ± 3.21	7.27 ± 3.86
**2014**	36.36	0	0	45.45	0	18.18
**2015**	47.82	4.34	4.34	26.08	4.34	13.04
**2014–15 average ± s.d.**	42.09 ± 8.10	2.17 ± 3.07	2.17 ± 3.07	35.77 ± 13.70	2.17 ± 3.07	15.61 ± 3.63
**2016**	21.73	0	0	39.13	4.34	34.78
**2017**	26.66	10	3.33	20	13.33	26.66
**2016–17 average ± s.d.**	24.20 ± 3.49	5.00 ± 7.07	1.67 ± 2.35	29.57 ± 13.53	8.84 ± 6.36	30.72 ± 5.74
**2018**	22.5	10	12.5	25	20	10
**2019**	24.32	8.10	21.62	13.51	21.62	10.81
**2018–19 average ± s.d.**	23.41 ± 1.29	9.05 ± 1.34	17.06 ± 6.45	19.26 ± 8.12	20.81 ± 1.15	10.41 ± 0.57
**2012–19 overall average ± s.d.**	32.42 ± 11.21	6.44 ± 4.39	7.72 ± 9.07	28.87 ± 10.19	8.52 ± 8.64	16.00 ± 10.07

s.d. = standard deviation.

**Table 4:** The percent (%) share of lag phases (from sampling to notification dates) in serious border rejection (sBR) notifications on food contact materials from the RASFF database (from 2012 until 2019) for all MS as an overall value and the top four MS that have contributed the highest numbers of sBR notifications.

Percent (%) Share of sBR Notifications					
Lag Phases (Days)	All RASFF MS (Overall)	Italy (IT)	Great Britain (GB)	Finland (FI)	Germany (DE)
**0–10**	6.42	18.75	5.55	0	0
**11–20**	18.34	34.37	16.66	0	33.33
**21–30**	21.10	31.25	22.22	18.75	25.00
**31–40**	8.25	3.12	5.55	18.75	0
**41–50**	13.76	9.37	5.55	31.25	0
**51–100**	18.34	3.12	22.22	25.00	33.33
**≥101**	11.00	0	22.22	0.00	8.33
**No sampling date recorded**	2.75	0	0	6.25	0
**Inverse date**	0	0	0	0	0
**Sampling date given as 1 January 1970**	0	0	0	0	0

**Table 5:** Percent (%) share of the hazards identified in sBR notifications in food contact materials (FCM) on a yearly basis (from the RASFF database between 2012 and 2019) and their biannual and overall averages.

Percent (%) Share of the Hazards Identified in sBR on FCM in RASFF (2012–2019)						
Year	Migration of PrimaryAromatic Amines	Migration of Formaldehyde	Migration of Formaldehyde and of Melamine	Migration of Metals	Migration of Melamine	Miscellaneous
	**sBR notifications**					
**2012**	37.5	12.5	0	50	0	0
**2013**	52.94	5.88	0	29.41	5.88	5.88
**2012–13 average ± s.d.**	45.22 ± 10.92	9.19 ± 4.68	0.00 ± 0.00	39.71 ± 14.56	2.94 ± 4.16	2.94 ± 4.16
**2014**	53.84	0	7.69	30.76	7.69	0
**2015**	44.44	22.22	0	33.33	0	0
**2014–15 average ± s.d.**	49.14 ± 6.65	11.11 ± 15.71	3.85 ± 5.44	32.05 ± 1.82	3.85 ± 5.44	0.00 ± 0.00
**2016**	50	0	8.33	33.33	0	8.33
**2017**	37.5	0	0	62.5	0	0
**2016–17 average ± s.d.**	43.75 ± 8.84	0.00 ± 0.00	4.17 ± 5.89	47.92 ± 20.63	0.00 ± 0.00	4.17 ± 5.89
**2018**	71.42	14.28	4.76	4.76	0	4.76
**2019**	66.66	9.52	4.76	14.28	4.76	0
**2018–19 average ± s.d.**	69.04 ± 3.37	11.90 ± 3.37	4.76 ± 0.00	9.52 ± 6.73	2,38 ± 3.37	2.38 ± 3.37
**2012–19 overall average ± s.d.**	51.79 ± 12.41	8.05 ± 8.11	3.19 ± 3.63	32.29 ± 18.21	2.29 ± 3.26	2.37 ± 3.42

s.d. = standard deviation.

## Data Availability

The data presented in this study are openly available in the European Commission RASFF portal database (https://webgate.ec.europa.eu/rasff-window/portal, accessed on 25 July 2020).
